# SCN2A Pathogenic Variants and Epilepsy: Heterogeneous Clinical, Genetic and Diagnostic Features

**DOI:** 10.3390/brainsci12010018

**Published:** 2021-12-24

**Authors:** Roberta Epifanio, Roberto Giorda, Maria Carolina Merlano, Nicoletta Zanotta, Romina Romaniello, Susan Marelli, Silvia Russo, Francesca Cogliati, Maria Teresa Bassi, Claudio Zucca

**Affiliations:** 1 Clinical Neurophysiology Unit, Scientific Institute IRCCS E. Medea, 23842 Bosisio Parini, LC, Italy; robiepifanio4@gmail.com (R.E.); nicoletta.zanotta@lanostrafamiglia.it (N.Z.); romina.romaniello@lanostrafamiglia.it (R.R.); susan.marelli@lanostrafamiglia.it (S.M.); 2 Molecular Biology Laboratory, Scientific Institute IRCCS E. Medea, 23842 Bosisio Parini, LC, Italy; roberto.giorda@lanostrafamiglia.it (R.G.); mariateresa.bassi@lanostrafamiglia.it (M.T.B.); 3 Royal University Hospital, University of Saskatchewan, Saskatoon, SK S7N 0W8, Canada; merlanomc@gmail.com; 4 Cytogenetics and Molecular Genetics Laboratory, Istituto Auxologico Italiano IRCCS, 20145 Milan, MI, Italy; s.russo@auxologico.it (S.R.); f.cogliati@auxologico.it (F.C.)

**Keywords:** genetics, epilepsy, *SCN2A* gene, autistic spectrum disorder, epileptic encephalopathy

## Abstract

Pathogenic variants of the *SCN2A* gene (MIM 182390) are associated with several epileptic syndromes ranging from benign familial neonatal-infantile seizures (BFNIS) to early infantile epileptic encephalopathy. The aim of this work was to describe clinical features among five patients with concomitant *SCN2A* gene variants and cryptogenic epileptic syndromes, thus expanding the *SCN2A* spectrum of phenotypic heterogeneity. *De novo* variants were identified in four patients, while one inherited variant was identified in a patient with an unaffected carrier biological father with somatic mosaicism. Two of five patients were diagnosed with a neonatal epileptic encephalopathy. The remaining three patients manifested a focal epileptic syndrome associated with autistic spectrum disorders (ASD) or with a variable degree of intellectual disability (ID), one of them displaying a hitherto unreported atypical late onset epilepsy. Overall, the pattern of clinical manifestations among these patients suggest that any observed neurological impairment may not be directly related to the severity of the electroclinical pattern, but instead likely associated with the mutation itself. Moreover, our results highlight the importance of *SCN2A* mutational screening in cases of ID/ASD with or without epilepsy.

## 1. Introduction

The *SCN2A* gene (MIM 182390) plays an important role in the initiation and propagation of action potentials [1]. It is responsible for encoding the Navα1.2 sub-unit of voltage-gated sodium channels. These channels are highly expressed in the adult central nervous system, particularly in excitatory glutamatergic neurons of the cortex and in both excitatory and inhibitory cells of the hippocampus [2,3].

Genetic mutations resulting in changes to this sodium channel protein have been associated with a broad spectrum of epileptic syndromes. In fact, the spectrum of clinical pictures associated with *SCN2A* mutations has expanded over the last decade. Common manifestations range from benign neonatal infantile seizures to severe epilepsy subtypes accompanied by intellectual disability (ID) and/or autistic traits with or without epilepsy [2,4,5,6]. Better characterization of the genotype–phenotype correlations is important as this has an impact on both genetic counseling and appropriate pharmacological therapies.

Recent reviews by Sanders and Spratt have led to advancements in the area with the development of models to help classify the broad spectrum of *SCN2A* phenotypes. They have proposed at least three classification groups: (1) neonatal and infantile epileptic encephalopathies, (2) benign infantile seizures, and (3) ASD/ID with developmental delay primarily of language and social milestones, with or without epilepsy [2]. Interestingly, neonatal and infantile epileptic encephalopathies were associated with missense mutations leading to increased sodium channel activity, thus referred to as gain-of-function mutations. Conversely, the ASD/ID category was associated with truncated or splice mutations leading to impaired protein function and diminished channel activity, thus referred to as loss-of-function mutations.

Despite these advances, however, emerging new clinical presentations cannot be adequately described by these prior models, suggesting the need for adjustment to prior classification schemes.

Here, we report a series of five patients with distinct *SCN2A* mutations, at least one of which adopting an unusual form of inheritance and an atypical onset of epilepsy not yet reported in the literature.

## 2. Materials and Methods

A total of 1800 patients with epileptic syndromes were followed by the Clinical Neurophysiological Unit of IRCCS E. Medea Epilepsy Center. Of these 1800 patients, 480 were determined to have a cryptogenic epileptic syndrome. Cryptogenic epileptic syndrome was defined as epilepsy of unclear etiology with a negative Microarray-based Comparative Genomic Hybridization (aCGH) test.

Of these 480 patients with cryptogenic epilepsy, 220 underwent genetic testing using a Next-Generation Sequencing (NGS) epilepsy panel comprising the exon-coding portions of 197 genes (see list in Supplementary Table S1). Libraries were synthetized with SureSelect QXT (Agilent, AGILENT, Santa Clara, CA, USA) and sequenced for 2 × 150 cycles on a NextSEq 500 (Illumina, Milano, Italy). All protein coding regions and at least 10 bases of each adjacent intronic sequence were analyzed with BWA enrichment software (Illumina, Milano, Italy). Minimum accepted coverage was 100×. Variants with minor allele frequency >1%, synonymous variants, and variants cited in ClinVar (www.ncbi.nlm.nih.gov/clinvar/, accessed on 15 October 2021) as Benign or Likely benign were excluded. Potential pathogenicity, according to the American College of Medical Genetics (ACMG) guidelines, was assessed with Varsome (varsome.com, accessed on 8 October 2021), as well as by consulting the dbSNP (ftp.ncbi.nih.gov/snp, accessed on 15 October 2021), ClinVar (www.ncbi.nlm.nih.gov/clinvar/, accessed on 15 October 2021) and HGMD (www.hgmd.cf.ac.uk/, accessed on 15 November 2021) databases.

Patient data on epilepsy symptomatology were analyzed in detail. Age of seizure onset, type of seizure, epileptic syndrome, and response to anti-epileptic treatment were recorded. All electroencephalogram (EEG) recordings were reviewed and classified according to the characteristics of background activity during wakefulness and sleep, and the presence of slow and epileptiform abnormalities were noted. All patients had a brain magnetic resonance imaging (MRI) performed either on a 1.5 T or 3 T MRI scanner. Written informed consent was obtained from all probands and their parents, and blood samples were obtained from each patient–parents trio. Refer to Table 1 for a summary of the clinical and EEG data of the five subjects.

### Case Series

**Case 1:** A 13-year-old boy born to healthy, unrelated parents after an uneventful pregnancy. Delivery was by cesarean section due to breech presentation. The recorded Apgar score was 10/10.

Seizures began early in the second day of life and required transfer to the neonatal intensive care unit. The patient was initially treated with phenobarbital (PB), but due to poor seizure control, phenytoin (PHT) was added to therapy. Fluctuating blood concentrations of PHT, however, led to gradual substitution with topiramate (TPM). Ultimately, a diagnosis of Ohtahara-like early epileptic encephalopathy was made and adrenocorticotropic hormone (ACTH) was added to therapy [7], resulting in a temporary reduction in seizure frequency.

At one year of age, spastic tetraparesis and complex visual impairment were noted. Brain MRI revealed a generalized reduction in brain volume with predominant white matter involvement, suggesting possible metabolic disease. Investigations, including skin and muscle biopsies, were non-diagnostic.

At 19 months, the patient’s epilepsy syndrome was characterized by clusters of focal seizures with head deviation, fencer posturing, ocular clonus, pallor, and unnatural laughter. Seizures were most often associated with a febrile illness and occurred on a monthly basis. The EEG demonstrated slowing of background activity and epileptic abnormalities prevalent in the temporal regions.

At the age of 5 years, PB was substituted with levetiracetam (LEV). At the age of 10, startle-like episodes began and TPM was replaced with sodium valproate (VPA). The patient underwent extensive genetic testing (Array-CGH, *STXBP1*, *SCN1A*, and *KCNQ2/KCNQ3* mutation analysis), all of which were negative.

**Figure 1:**
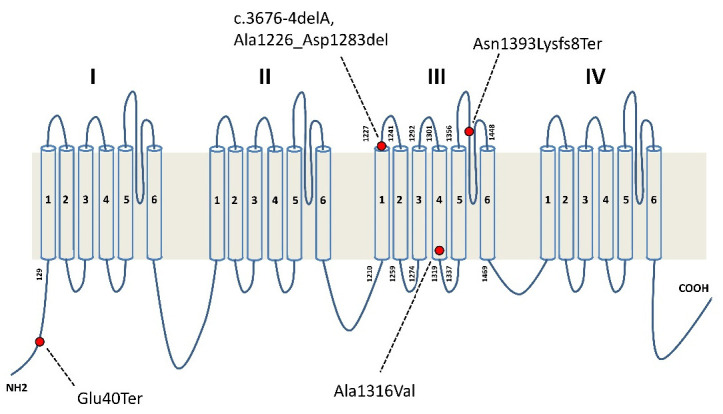
Location of *SCN2A* mutations identified in our patients.

At the age of 11 years, analysis with a specific epilepsy genes panel revealed the following *SCN2A* gene *de novo* variant: NC_000002.11:g.166229832 C>T, NM_021007.2:-c.3947C>T (p. Ala1316Val) (rs796053130). Notably, this variant has been previously reported in the literature in two patients, including one with Ohtahara syndrome [8,9] (Figure 1).

**Case 2:** A 27-year-old male born to healthy, unrelated parents after an uneventful pregnancy and delivery. A predominance of clonic seizures began in the second day of life and led to a diagnosis of Early Myoclonic Encephalopathy. Seizures were resistant to treatment with ACTH and Felbamate (FLB).

The patient’s epilepsy syndrome was characterized by daily polymorphic, drug-resistant seizures. Neurological examination revealed spastic tetraparesis with axial hypotonia. Metabolic tests were negative.

EEG recordings up to the age of 3 months were characterized by a burst-suppression pattern. Thereafter, EEG demonstrated poorly organized and high voltage background activity with subcontinuous diffuse paroxysmal slowing and epileptiform abnormalities. These were most prevalent over the occipital regions. Brain MRI demonstrated: slight enlargement of the subarachnoid spaces at 2 months; diffuse reduction in white matter volume at 12 months; and reduction in corpus callosum volume and signal abnormalities within peritrigonal areas and semi-oval centers at 4 years.

At the age of 27 years, analysis with a specific epilepsy genes panel revealed the *SCN2A* gene *de novo* variant NC_000002.11:g.166229832 C>T, NM_021007.2:-c.3947C>T (p. Ala1316Val) (rs796053130). This is the same variant identified in Case 1.

**Case 3:** A 9-year-old female born to healthy, unrelated parents after an uncomplicated pregnancy and delivery. The patient demonstrated delayed speech development and behavioral abnormalities that led to a diagnosis of autism spectrum disorder (ASD) in her first year of life. Her intelligence quotient (IQ) at the age of 2.5 years was 38 according to the Griffith Scale.

**Figure 2:**
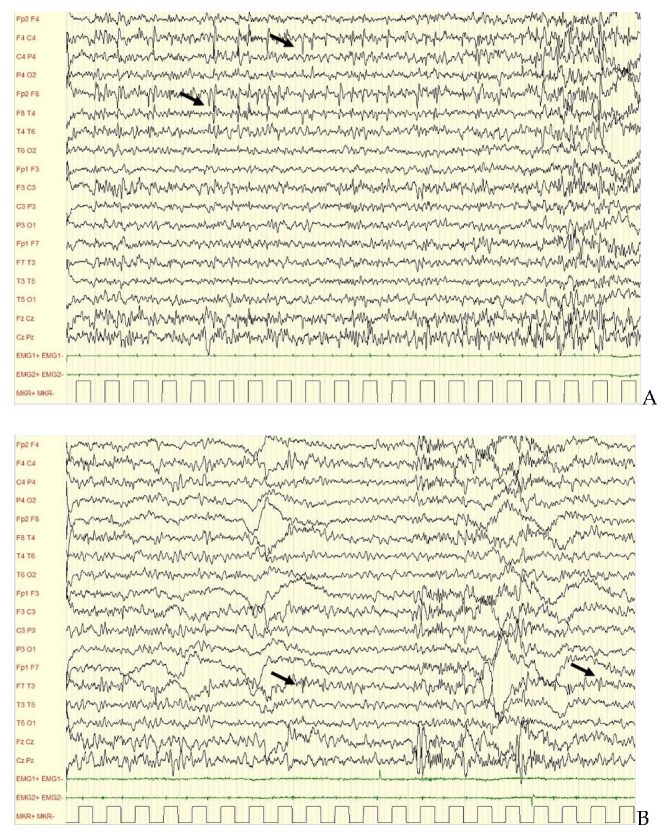
Case 3, polysomnographic EEG showing epileptiform abnormalities (arrows) over right centrotemporal regions (**A**) and over left frontotemporal regions (arrows) (**B**) in two different recordings during follow up.

Seizure onset began at 2 years of age. Seizures occurred while awake and were characterized by staring and rhythmic cough that culminated in sleep. The EEG demonstrated irregular background activity with epileptiform abnormalities over centrotemporal and frontotemporal regions with alternating side predominance (Figure 2). Brain MRI, metabolic tests and Array-CGH were all negative.

At the age of 2 years, VPA treatment was initiated and remission was achieved. At the age of 6 years, a reduction in blood VPA levels led to paroxysmal events with impaired consciousness, abnormal eye deviation, and impaired sleep. Seizure control was again achieved with increased VPA dosing.

At the age of 9 years, the patient was screened with an NGS epilepsy genes panel revealing a heterozygous *de novo SCN2A* splice site variant: NC_000002.11 g.-166226632delA, NM_021007.2:-c.3676-4delA (p.Ala1226_Asp1283del). This splicing error leads to an in-frame deletion that generates a truncated protein lacking 58 amino acids (corresponding to exon 20). This results in loss of the S2 helix and its extracellular upstream loop, a large segment of the S3 helix, and the intracellular loop between S2 and S3 on the third protein domain (Figure 1). Degradation of mRNA as a result of the mutation cannot be excluded, thus resulting in cessation of protein production and haploinsufficiency.

**Case 4:** An 18-year-old female born to unrelated parents after an uneventful pregnancy and delivery. The patient demonstrated early delayed psychomotor development, particularly in the language domain. This severe speech delay, in combination with the limited and repetitive behavior patterns, were consistent with a diagnosis of ASD. At the age of 12, the Stanford–Binet intelligence test revealed severe mental retardation (IQ less than 30).

Seizure onset began at the age of 8 years, and was characterized by daily seizures of a few second durations. They were associated with sudden awakening, wheezing, stiffening, and incontinence. VPA treatment was initiated resulting in a significant reduction in seizure frequency, with only a few breakthrough seizures during febrile illness.

The first EEG was characterized by normal background activity with slow waves over the right frontal regions during both wake and sleep states. MRI and genetic testing results (karyotype, Array-CGH) were normal.

Unfortunately, at the age of 14, the patient’s neurological condition worsened and she began to have seizures on a weekly basis. During this period, her EEG revealed epileptiform abnormalities in the right temporal lobe, with a predominance in the sleep state. Clobazam (CLB) therapy was thus initiated at bedtime. Although this resulted in a reduction in seizure frequency, the patient continued to experience monthly seizures.

At the age of 14 years, epilepsy NGS genetic panel testing revealed a *de novo SCN2A* 4 bp deletion (NC_000002.11g.166231398_166231401del, NM_021007:c.4176-4179delCAAT; p.Asn 1393LysfsTer8). This mutation results in premature termination of protein synthesis, with loss of the fourth protein domain and of part of the third downstream extracytoplasmic loop between segments S5 and S6 (Figure 1).

It was not until the age of 14 when the *SCN2A* genetic variant was identified that appropriate therapy with a sodium channel blocker (Carbamazepine) was initiated, resulting in complete seizure remission.

**Case 5:** A 36-year-old male born to healthy, unrelated parents after an uneventful pregnancy and delivery. From early life, the patient demonstrated delayed psychomotor development, primarily in the language domain. The neurological examination was positive for severe dyspraxia, bradykinesia, diffuse brisk tendon reflexes, and intellectual disability (maximum IQ of 40 at 15 years of age).

From the age of 9 years, EEG recordings revealed asynchronous epileptiform abnormalities over the central regions bilaterally, but no seizures were reported. Epilepsy started at the age of 26, and was characterized by tonic seizures that were preceded by a scream and occurred during the sleep.

The most recent EEG recordings showed slow and epileptiform focal abnormalities with a predominance over the right frontal regions. MRI 3T showed slight bilateral cerebellar hemispheric atrophy. Metabolic, imaging and genetic testing (karyotype, fragile X syndrome, sub-telomeric rearrangements, Array-CGH) were negative.

Seizure remission was achieved with CBZ therapy in combination with CLB at bedtime. At the age of 35 years, NGS epilepsy genes panel analysis revealed a heterozygous nonsense *SCN2A* gene mutation that was also identified in the patient’s asymptomatic carrier father with determined somatic mosaicism (approximately 50%) (NC_000002.11:g.166152451G>T, NM_021007.2:c.118G>T (p.Glu40Ter). This mutation results in a premature stop codon where the mRNA undergoes nonsense-mediated decay (NMD).

## 3. Results

Screening of 220 patients using an NGS epilepsy panel revealed a total of 9 patients with SCN2A gene variants. Five of these patients carried pathogenic variants while the remaining four were of uncertain significance.

Table 2 summarizes variants characteristics according to HGVS nomenclature. Non-pathogenic variants are listed in Supplementary Table S2.

Cases 1 and 2 in our series can be classified as neonatal epileptic encephalopathies with severe neurodevelopmental impairment and abnormal brain imaging. This pathogenic variant is characteristic of a gain-of-function mutation.

Cases 3 and 4 presented with a similar clinical phenotype characterized by ADS/ID and a genotype resulting in truncated proteins. Only slight differences were noted in regard to age of onset, response to treatment, and evolution of EEG pattern. Interictal EEG recordings in both patients demonstrated a frontotemporal focus, with Case 3 exhibiting epileptiform abnormalities with alternating side predominance (Figure 2A,B). This can sometimes be observed in genetically determined epilepsies [10].

Case 5 was unique in that the patient presented with late onset epilepsy, and an SCN2A genotype inherited from a father with somatic mosaicism. Late onset epilepsy has only been reported once in the literature [11]. Moreover, the early truncating variant is uncommon in the genetic databases (https://databases.lovd.nl/shared/genes/SCN2A, accessed on: 15 October 2021).

According to the bio-informatic predictions, three of four variants (Cases 3–5) generate a truncated protein. The pathogenic variants of Cases 3 and 5 may be associated with mRNA degradation due to NMD, resulting in SCN2A protein haploinsufficiency.

## 4. Discussion

Numerous pathogenic variants of the sodium channel *SCN2A* gene have been identified in patients with epilepsy and intellectual disability. As a result, there is a broad range of clinical phenotypes. Although some advancement has been made in regard to the classification of these phenotypes [2,12], this case series and more recent literature have demonstrated a broader spectrum of manifestations that requires adjustment to our prior classification models [13].

Cases 1 and 2 can be classified as neonatal epileptic encephalopathies with severe neurodevelopmental impairment, as previously described by Sanders’ *SCN2A* Classification Model [2]. The associated gain-of-function mutations result in increased sodium channel activity, potentiating neuronal excitability and, thus, explaining the epileptic phenotype. Importantly, for these two cases, late genetic testing resulted in late initiation of the appropriate anti-epileptic therapy. This highlights the importance of an early genetic diagnosis.

On the other hand, Cases 3 and 4 can be classified as ASD/ID with developmental delay with or without epilepsy, as described by the aforementioned Classification Model [2]. In keeping with this model, these *SCN2A* pathogenic variants result in loss-of-function mutations that diminish sodium channel activity, and have been associated with a phenotype predominantly characterized by ASD/ID. Notably, Cases 3 and 4 demonstrated good response to treatment, with complete seizure remission achieved in Case 4 only after initiation of a sodium channel blocker. This observation differs in part from prior literature which supports better treatment efficacy in gain-of-function mutations.

Notably, the electroclinical pictures of Cases 3 and 4 cannot be considered age related, nor can they be classified as epileptic encephalopathies. Considering ASD/ID diagnosis prior to seizure onset, together with mild seizure severity and good response to treatment, the neuropsychological impairment cannot be solely attributed to the epileptic syndrome. Instead, the mutation itself is a likely contributor to the electroclinical picture. Therefore, *SCN2A* encephalopathy should be considered within the group of Developmental and Epileptic Encephalopathies. It represents a complex neurodevelopmental disorder where both ID and epilepsy play a synergic role in the phenotype evolution [14,15,16].

Furthermore, the coexistence of epilepsy and ASD may be a multifactorial process affecting large-scale brain networks and their development. This may result in abnormal cortical development or focal regions of abnormal cytoarchitecture [17]. However, these last features are often difficult to identify as they are not easily detected by clinical MRI scans.

The fifth patient presented with an atypical late-onset focal epileptic syndrome not previously reported in the literature. The late seizure onset again suggests that neurological impairment is likely mediated more by the pathogenic variant than by electroclinical pattern severity. Moreover, the presence of a very early truncating variant, likely leading to haploinsufficiency, further supports the concept that loss of function mutations have ID as their main clinical manifestation. Furthermore, the mosaicism detected in the asymptomatic father lends support to genetic counseling prior to conception, as this has been previously observed with other genes associated with epileptic syndromes [18,19,20]. Indeed, carrier parents with a low degree of mosaicism are easily missed, lending support to diagnostic tools such as NGS genetic testing with higher sensitivity for genetic risk.

## 5. Conclusions

This case series illustrates that disruptions in the *SCN2A* gene can be associated with diverse clinical pictures of epilepsy and altered neuropsychological function. New presentations of these phenotypic variants require us to expand our earlier classification models. Moreover, the resultant electroclinical impairments observed in patients appear to have a direct correlation with the genetic mutation, rather than the resulting epileptic syndrome’s severity. Finally, the utility of early NGS genetic testing has been demonstrated in ASD/ID cases without epilepsy, as well as in genetic counselling among unaffected carrier parents.

## Figures and Tables

**Table 1:** Clinical and EEG data of patients with *SCN2A* pathologic variants.

Case	Clinical Picture	Onset of Neurological and Neuropsychological Impairment	Epilepsy Onset	Circadian Seizure Incidence	EEG	Effective AEDS
1	EE Ohtahara-like, spastic tetraparesis, CVI, severe ID.	First year of life	Second day of life (from 19 months: mainly focal seizures)	Both during wakefulness and sleep	Initially BSP, then SBA, EA predominant over temporal areas.	TPM, VPA
2	Early Myoclonic Encephalopathy, spastic tetraparesis, severe ID.	First year of life	Second day of life	Both during wakefulness and sleep	Initially BSP, then POBA. Subcontinous SWA and EA predominant over occipital areas.	Drug resistance
3	ASD, ID—medium ID, focal epilepsy	First year of life	2 years of life	Mainly during wakefulness	IBA, EA over frontotemporal areas bilaterally.	VPA
4	ASD, severe ID, focal epilepsy	First year of life	8 years of life	Rare during wakefulness, mainly during sleep.	NBA, SA over right frontal areas. EA over right temporal areas.	Initially VPA, then CBZ (after 14 years of life)
5	Medium ID, focal epilepsy (late onset)	Second year of life	26 years of life	During sleep	NBA, SA and EA predominant over right frontal areas.	CBZ + CLB

EE: Epileptic Encephalopathy, CVI: Complex Visual Impairment, ID: Intellectual Disability, ASD: Autism Spectrum Disorder. BSP: burst-suppression pattern, NBA: normal background activity; SBA: slowing of background activity; IBA: irregular background activity; POBA: poorly organized background activity. SA: slow abnormalities; EA: epileptiform abnormalities. TPM: topiramate, VPA: sodium valproate, CBZ: carbamazepine, CLB clobazam.

**Table 2:** Clinical and genetic features of *SCN2A* mutated patients.

Case	Clinical	Mutation Type	Mutation	Origin
1	Typical EIEE related to SCN2A mutation	Missense	p.Ala1316Val	*De novo*
2	Typical EIEE related to SCN2A mutation	Missense	p.Ala1316Val	*De novo*
3	ASD, low IQ, focal epilepsy	Splice site	p.Ala1226_Asp1283del	*De novo*
4	ASD, low IQ, focal epilepsy	Frameshift	c.4176-4179delCAAT; p.Asn1393LysfsTer8	*De novo*
5	Low IQ, late onset focal epilepsy	Nonsense	p.Glu40Ter	From mosaic father

EIEE: early infantile epileptic encephalopathy; ASD: autism spectrum disorder; IQ: intelligence quotient.

## Data Availability

Data available on request due to ethical restrictions.

## Supplementary Materials

The following supporting information can be downloaded at: https://www.mdpi.com/article/10.3390/brainsci12010018/s1, Table S1: List of genes (in alphabetical order) of NGS Epilepsy panel; Table S2: List of non-pathogenic variant.
