# Supplementary material for: SCN2A Pathogenic Variants and Epilepsy: Heterogeneous Clinical, Genetic and Diagnostic Features
Source: Brain Sci. 2021 Dec 24;12(1):18. doi: 10.3390/brainsci12010018 (PMC8773615; doi:10.3390/brainsci12010018)
Supplement: Supplementary file 1 [file brainsci-12-00018-s001.zip › Supplementary File S1.pdf]

Table S1 - List of genes (in alphabetical order) of NGS Epilepsy panel

AARS, ADGRV1, ADRA2B, ADSL, ALDH4A1, ALDH7A1, ALG13, ALPL, ARHGEF15, ARHGEF9, ARX, ASAH1, ATP1A2, ATP1A3, ATP6V1A, BRD2, CACNA1A, CACNA1G, CACNA1H, CACNA2D2, CACNB4, CBL, CDKL5, CERS1, CHD2, CHRNA2, CHRNA4, CHRNA7, CHRNB2, CLCN2, CLCN4, CLN3, CLN5, CLN6, CLN8, CLTC, CNKSR2, CNTNAP2, CPA6, CPLX1, CSNK1G1, CSNK2B, CTNND2, CUX2, DEPDC5, DHDDS, DNM1, DNM1L, DOCK7, DYNC1H1, EEF1A2, EFHC1, EIF2S3, EMC1, EPM2A, FASN, FLNA, FOXG1, GABBR2, GABRA1, GABRA2, GABRA3, GABRA5, GABRB2, GABRB3, GABRD, GABRG2, GAL, GAMT, GNAO1, GOSR2, GRIA1, GRIN1, GRIN2A, GRIN2B, HCN1, HCN2, HCN4, HDAC4, HNRNPU, IDH3A, IQSEC2, JRK, KANSL1, KCNA1, KCNA2, KCNB1, KCNC1, KCND2, KCND3, KCNH1, KCNH5, KCNJ10, KCNMA1, KCNQ2, KCNQ3, KCNT1, KCTD7, KIAA2022, KPNA7, KPTN, LGI1, LMNB2, MBD5, MDH2, MECP2, MEF2C, MFSD8, MICAL1, MTOR, NACC1, NAPB, NECAP1, NEDD4L, NHLRC1, NHLRC2, NPRL2, NPRL3, NRXN1, NTRK2, NUS1, OTUD7A, PACS2, PCDH12, PCDH19, PIGA, PIGC, PIGN, PIGO, PIGP, PIGQ, PIGV, PLCB1, PNKP, PNPO, POLG, PRICKLE1, PRIMA1, PROSC, PRRT2, PTPN23, PURA, QARS, RAB11A, RBFOX1, RBFOX3, RHOBTB2, ROGDI, RYR3, SCARB2, SCN10A, SCN10A, SCN1A, SCN1B, SCN2A, SCN3A, SCN8A, SCN9A, SEPSECS, SETD1B, SETD2, SIK1, SLC12A5, SLC13A5, SLC25A22, SLC2A1, SLC35A2, SLC6A1, SLC6A9, SLC9A6, SMC1A, SMS, SNAP25, SPTAN1, SRPX2, ST3GAL3, STX1B, STXBP1, SYN1, SYNGAP1, SZT2, TBC1D24, TBCD, TCF4, TNK2, TPP1, TRAK1, TRAPPC6B, TSC1, TSC2, UBA5, UBE3A, UNC80, WDR45, WWOX, YWHAG, ZDHHC9, ZEB2.
