# Supplementary material for: SCN2A Pathogenic Variants and Epilepsy: Heterogeneous Clinical, Genetic and Diagnostic Features
Source: Brain Sci. 2021 Dec 24;12(1):18. doi: 10.3390/brainsci12010018 (PMC8773615; doi:10.3390/brainsci12010018)
Supplement: Supplementary file 1 [file brainsci-12-00018-s001.zip › Supplementary File S2.pdf]

Table S2 - List of non-pathogenic variant

Case	Gene	Chr	Coordinate	Genotype	dbSNP ID	Type	Sift	PolyPhen	HGVSc	HGVSp	Frequency (ExAc)	Inheritance
6	SCN2A	2	166172021	het	.	nonsynonymous SNV	Damaging	Benign	NM_021007.2:c.1424G>A	NP_066287.2:p.p.Ser475Asn		Paternal
7	SCN2A	2	166172021	het	.	nonsynonymous SNV	Damaging	Benign	NM_021007.2:c.1424G>A	NP_066287.2:p.p.Ser475Asn		Paternal
8	SCN2A	2	166221662	het	rs1204126053	nonsynonymous SNV	Tolerated	Benign	NM_001040142.1:c.3409G>T	NP_001035232.1:p.Ala1137Ser	0,0008	Maternal
9	SCN2A	2	166231450	het	.	nonsynonymous SNV	Tolerated	Benign	NM_001040142.1:c.4228C>A	NP_001035232.1:p.Leu1410Ile		Maternal
